# High-security-level multi-dimensional optical storage medium: nanostructured glass embedded with LiGa_5_O_8_: Mn^2+^ with photostimulated luminescence

**DOI:** 10.1038/s41377-020-0258-3

**Published:** 2020-02-18

**Authors:** Shisheng Lin, Hang Lin, Chonggeng Ma, Yao Cheng, Sizhe Ye, Fulin Lin, Renfu Li, Ju Xu, Yuansheng Wang

**Affiliations:** 1 Key Laboratory of Optoelectronic Materials Chemistry and Physics, Key Laboratory of Design and Assembly of Functional Nanostructures, Fujian Institute of Research on the Structure of Matter, Chinese Academy of Sciences, Fuzhou, Fujian 350002 China; 2 University of Chinese Academy of Sciences, Beijing, 100049 China; 3 CQUPT-BRU Innovation Institute, Chongqing University of Posts and Telecommunications, Chongqing, 400065 China; 4 Xiamen Institute of Rare-earth Materials, Haixi Institutes, Chinese Academy of Sciences, Xiamen, Fujian 361000 China

**Keywords:** Optical materials and structures, Optical data storage

## Abstract

The launch of the big data era puts forward challenges for information preservation technology, both in storage capacity and security. Herein, a brand new optical storage medium, transparent glass ceramic (TGC) embedded with photostimulated LiGa_5_O_8_: Mn^2+^ nanocrystals, capable of achieving bit-by-bit optical data write-in and read-out in a photon trapping/detrapping mode, is developed. The highly ordered nanostructure enables light–matter interaction with high encoding/decoding resolution and low bit error rate. Importantly, going beyond traditional 2D optical storage, the high transparency of the studied bulk medium makes 3D volumetric optical data storage (ODS) possible, which brings about the merits of expanded storage capacity and improved information security. Demonstration application confirmed the erasable–rewritable 3D storage of binary data and display items in TGC with intensity/wavelength multiplexing. The present work highlights a great leap in photostimulated material for ODS application and hopefully stimulates the development of new multi-dimensional ODS media.

## Optics: Next generation of optical data storage systems

An optical data storage (ODS) system that is cost-effective to make and easily scalable could lay the foundations for data storage technologies with higher capacity and improved security. The era of big data is driving the need for data storage systems with larger capacity, better security and improved performance. However, even the latest two-dimensional optical disk technologies cannot store more than one terabyte of data, leading scientists to explore alternative technologies. Now, a team of Chinese researchers, led by Yuansheng Wang from the Chinese Academy of Sciences, has developed a three-dimensional ODS system that uses light-emitting nanocrystals (NCs) to store and retrieve information. Made from a transparent glass ceramic embedded with photostimulated luminescent rare-earth-doped metallic NCs, the new ODS system can store 3D optical data with increased capacity and improved security compared with current technologies.

## Introduction

Currently, the heritage of human civilization depends on the preservation of digitalized information, including character, image, audio and video, which spawns countless data. The resulting information explosion stimulates the continuous upgrade of the storage medium and mode. In this context, magnetic data storage has been gradually replaced by optical data with higher efficiency, lower energy consumption, larger capacity and longer service lifetime^1^.

However, state-of-the-art two-dimensional (2D) optic disks still have a bottleneck in storage capacity, making it difficult to go beyond 1 TB^2^. As such, two promising approaches, i.e., near/far-field super-resolution optical microscopy to overcome the optical diffraction limit^3–6^ and information multiplexing to expand the physical dimension of the optical data storage (ODS) medium^7–10^, were proposed and are under development. A prerequisite for these advanced technologies is to be capable of sophisticated manipulation of light–matter interactions at the nanoscale^2^. To date, several kinds of nanomaterials, such as metallic nanocrystals (NCs)^8,11^, graphene oxide^12–14^, semiconductor quantum dots^15–17^ and rare-earth ion-doped NCs^18,19^, have been developed as ODS media, utilizing their physical and/or chemical state variations during light–matter interactions. Despite the fascinating ODS properties, the shortcomings of high cost, tedious preparation route and low production greatly hinder their practical applications; moreover, these NCs are bound to disperse into organic hosts, which brings about a stability issue: colouration- or shrinkage-induced performance reduction under long-term repeatable laser write-in/read-out^9,20–22^. Evidently, the exploration of new ODS nanomaterials to address the issues stated above is able to promote rapid progress in information preservation technology.

As a classical kind of ODS medium, photostimulated (PSL) materials with persistent luminescence (PersL) have attracted researchers’ interest since their discovery because of their good erasable–rewritable ability and ultrafast writing speed^23–30^. The Quantex Corporation first evaluated its validity for ODS and extended the application to parallel Boolean logic operations and associative memory^23,24^. Recently, its prospect for ODS was demonstrated with the aid of bit-by-bit ultraviolet (UV)-/blue-laser encoding and global-scanned near-infra-red (NIR)-laser decoding^21,31–33^. Nevertheless, PSL materials are still not in consideration as an alternative to big data storage media due to their difficulties in achieving nanocrystallization—the light–matter interaction on the micrometre scale would result in a small writing/reading resolution to a great extent and thus limited storage capacity. There are two mutually contradictory aspects for attaining a nanosized PSL material: on the one hand, high temperature is required to induce suitable deep traps in the host responsible for the charging/releasing of charge carriers (electrons or holes) during data encoding/decoding and, on the other hand, high temperature is known to result in severe particle coarsening and agglomeration. Few examples have succeeded in achieving PSL in nanosystems^34,35^, let alone considered their viability for large-capacity ODS.

Herein, we develop a new kind of ODS medium, PSL transparent glass ceramic (TGC), via in situ precipitation of PSL LiGa_5_O_8_: Mn^2+^ NCs from a glass matrix. The controlled thermally driven glass crystallization leads to a highly ordered nanostructure in the glass network, while the self-limited growth of LiGa_5_O_8_: Mn^2+^ NCs facilitates the generation of deep defects for PSL at a relatively low temperature due to low ionic diffusion mobility and, thus, the balance between nanosized grains and PSL performance is leveraged. This bulk PSL material with robustness can be fabricated in a cost-effective, environmentally friendly and scalable way in one step. Importantly, we demonstrate an unprecedented multidimensional scheme with a high-security level in the developed transparent PSL ODS medium due to the expanded volume and intensity/wavelength multiplexing.

## Results

### Self-limited growth of PSL LiGa_5_O_8_: Mn^2+^ NCs in glass

**Fig. 1:**
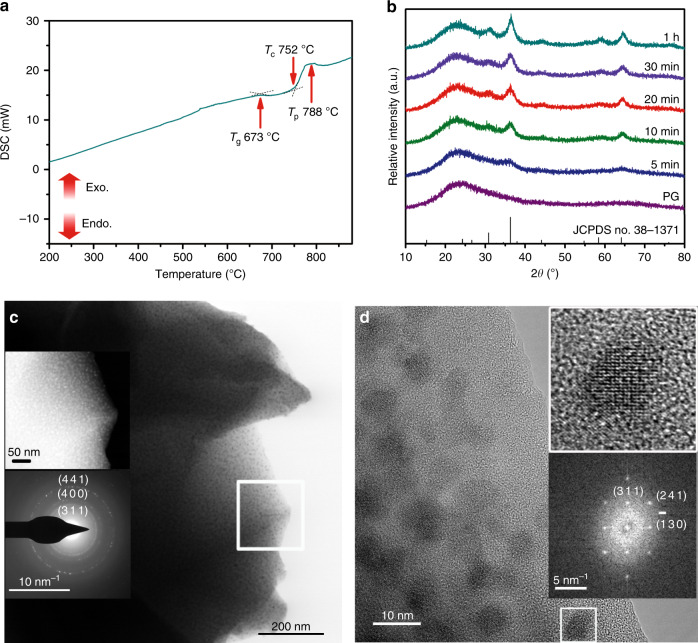
Controllable glass crystallization for in situ precipitation of LiGa_5_O_8_: Mn^2+^ NCs. **a** DSC curve of PG recorded at a heating rate of 10 K/min (*T*_g_, *T*_c_ and *T*_p_ denote the glass transition temperature and the onset and peak crystallization temperature, respectively). **b** XRD patterns of PG and TGC annealed at 750 °C for different durations. **c** Bright-field TEM observation of TGC; insets present HAADF-STEM microscopy on the selected region (top) and the SAED pattern (bottom). **d** HRTEM observation of the TGC; insets show the denoted nanoparticle with high magnification and the corresponding FFT pattern

The precursor glass (PG) with a stoichiometric composition (in mol%) of 68SiO_2_-7Al_2_O_3_-5Na_2_O-12.9Ga_2_O_3_-7Li_2_O-0.1MnO (abbreviated as SANGL glass) was fabricated via a melt-quenching route. Differential scanning calorimeter (DSC) analysis of PG indicated one exothermic peak (Fig. 1a). From the following X-ray diffraction (XRD) measurements (Fig. 1b), it is ascribed to cubic LiGa_5_O_8_ nanocrystallization (JCPDS NO. 38–1371). As revealed by the derived parameters from the DSC curves, the studied material has a favourable capacity to realize controllable nucleation and crystal growth during heat treatment (Supplementary Note 1)^36,37^. The glass crystallization proceeds by annealing PG at the onset of the crystallization temperature (750 °C) for various durations. With the annealing time prolonged from 5 min to 1 h, the gradual phase transformation from amorphous to crystalline occurs and the size of the precipitated LiGa_5_O_8_ NCs increases from ~2 nm to ~7 nm, according to the Scherrer equation. Transmission electron microscopy (TEM) accompanied by selected area electron diffraction shows the amorphous nature of PG (Supplementary Fig. S1) and after crystallization the monodispersed NCs in the glass matrix with clear polycrystalline concentric rings correspond to the (311), (400) and (441) facets of LiGa_5_O_8_ (Fig. 1c). High-angle annular dark-field scanning TEM (top inset of Fig. 1c) clearly distinguishes the contrast between LiGa_5_O_8_ (bright) and the aluminosilicate glass matrix (dark) due to the large difference in atomic number: Ga (*Z* = 31) vs. Al/Si (*Z* = 14/13). High-resolution TEM observations reveal several monodispersed LiGa_5_O_8_ NCs with different crystallographic orientations (Fig. 1d). For one nanoparticle marked by a white box, the fast Fourier transform pattern shows diffraction along the [31$\overline {10}$] zone axis. The measured angle between the (311) and ($\bar 1$30) facets is 90.0° and that between the (311) and (241) facets is 43.6°, both of which are close to the theoretical values. The above results firmly demonstrate that the highly ordered nanostructure of LiGa_5_O_8_-embedded TGC is favourable for photonic applications.

**Fig. 2:**
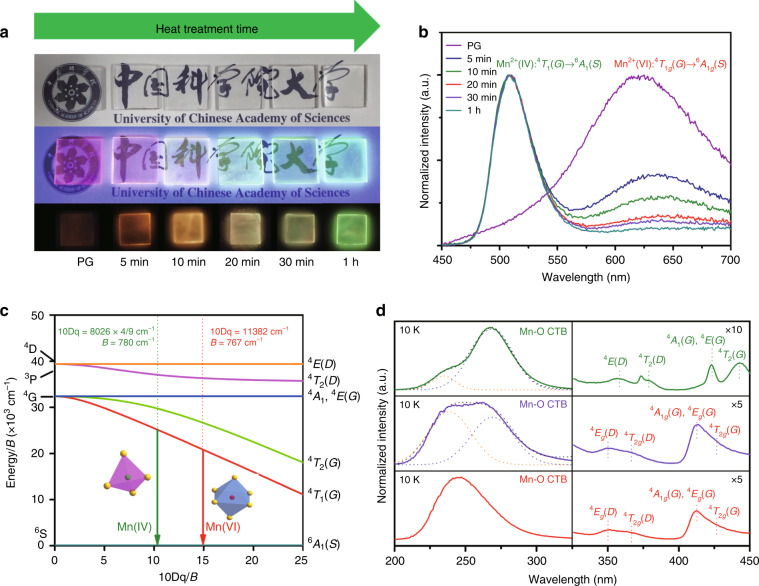
Site variation of Mn^2+^ after glass crystallization induces multicolour emissions. **a** Digital photographs of PG and TGCs under natural light (top), 254 nm UV light (middle) and thermal stimulation (bottom); in the condition of thermal stimulation, all the samples were charged by 254 nm light in advance for 5 min, delayed for 10 min to eliminate PersL and then heated to 150 °C (for TGC) or 500 °C (for PG). **b** Normalized photoluminescence spectra of PG and TGCs at room temperature, reflecting colour variation as glass crystallization proceeds. **c** Tanabe–Sugano diagram of 3*d*^5^ electronically configured transition metal ion, showing the influence of crystal field strength on energy levels of Mn^2+^. **d** PLE spectra of PG and TGC measured at 10 K

Glass can be perceived as a ‘supercooled liquid’ in a thermodynamically metastable state, so it tends to crystallize upon the supply of appropriate thermal energy. However, controlled glass crystallization remains arduous, as nucleation and growth stages usually overlap with each other^38^. Only when the nucleation rate is boosted and the growth rate is suppressed can ordered crystal-in-glass nanocomposites with high transparency be obtained, as in the present case (Fig. 2a and Supplementary Fig. S2). The transmittance in the visible region is higher than 70% for all TGCs with a thickness of 2 mm. It is believed that the heterogeneous structure of SANGL glass on the nano/mesoscale plays a key role in controlled glass crystallization, similar to previous reports in other oxide glass systems^39–42^. The ‘fragile’ part of SANGL glass shares a homologous chemical composition with the initial crystalline phase (and possibly, even topological crystalline-like ordering exists), which induces explosive nucleation of LiGa_5_O_8_ throughout the bulk glass upon heating. In contrast, the ‘strong’ part of SANGL glass with strong chemical bonding hardly nucleates, but it serves as a diffusion barrier to limit further crystal growth. A schematic illustration of the self-limited growth of LiGa_5_O_8_: Mn^2+^ NCs in SANGL glass is presented in Supplementary Fig. S3.

### Site occupation analyses of optically active Mn^2+^

The spectroscopic studies reveal the variation of local chemistry for the optically active Mn^2+^ ions before/after crystallization. Under 254 nm UV light excitation, a broadband emission is found with a maximum at 625 nm in PG and an additional narrow band with a peak at 510 nm in TGC (Fig. 2b). Both bands are ascribed to the Mn^2+^: ^4^T_1_(G) → ^6^A_1_(S) transition, but in different coordination environments, viz. octahedron (Mn(VI)) and tetrahedron (Mn(IV)), respectively^43,44^. The Tanabe–Sugano diagram in Fig. 2c reflects the variation in the energy levels of Mn^2+^ with the 3*d*^5^ electronic configuration sensitive to local perturbation. The crystal field strength (10Dq) and Racah parameter (B) are determined to be 3567 cm^−1^ and 780 cm^−1^ for Mn(IV) in the LiGa_5_O_8_ NCs, and 11382 cm^−1^ and 767 cm^−1^ for Mn(VI) in glass, respectively (Supplementary Note 2)^45,46^. With prolonged heat treatment time, the green emissive component intensifies with the compensation of the red component, so the visual colour is transformed in the range of red to green (Fig. 2a). Photoluminescence (PL) spectra measured at 10 K provide more information not observed at room temperature (Supplementary Fig. S4), where the detected additional emission signals possibly come from the defect centre and Mn^2+^–Mn^2+^ dimers^47,48^. PL excitation spectra at 10 K show the typical Mn^2+^: 5*d* → 5*d* transitions and Mn^2+^–O^2−^ charge transfer band (CTB). The spectral profiles monitoring green and red emissions are totally different from each other, confirming that Mn^2+^ ions are situated in different coordination environments.

### PSL performance and mechanism

**Fig. 3:**
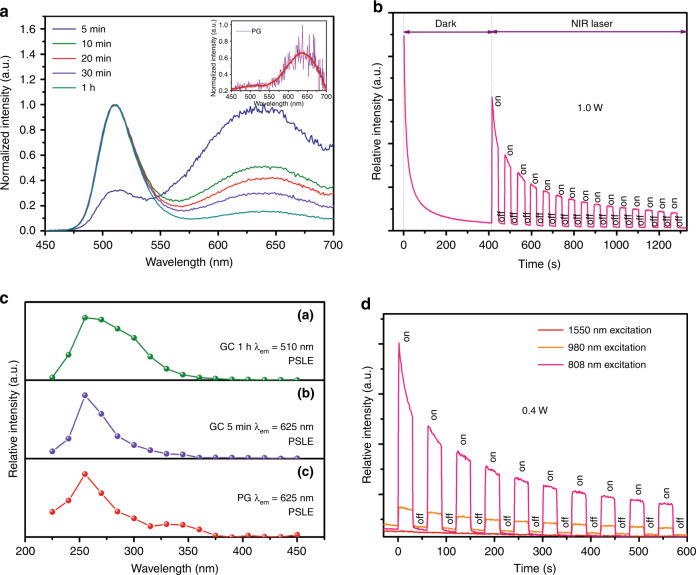
PSL performance of the LiGa_5_O_8_: Mn^2+^ NC-embedded TGC. **a** PSL spectra of PG and TGC heat treated at 750 °C for different durations. **b** PSL decay curve (*λ*_em_ = 510 nm) of TGC recorded by periodically controlling the on/off state of a 1 W 808 nm NIR laser (the on/off intervals set as 30 s). Before NIR stimulation, the sample was preirradiated at 254 nm for 5 min and naturally decayed to show no PersL in the dark. **c** The created PSL excitation (PSLE) spectra for PG and TGC. **d** PSL decay curves (*λ*_em_ = 510 nm) of TGC recorded in a 30 s periodic mode stimulated by 808, 980, and 1550 nm NIR lasers (power set as 0.4 W). Note: the charging condition is the same as that in **b**

Remarkably, in stark contrast to previous cases^21,22,33,49^, where only the submicrometre- or micrometre-sized phosphors yield PSL, the nanostructured LiGa_5_O_8_: Mn^2+^-embedded TGCs show good PSL performance and, interestingly, the PSL colour tone is tunable depending on the crystallization degree (Fig. 3a and Supplementary Fig. S5). The active centres undoubtedly can be ascribed to Mn^2+^ due to the similar spectral profiles of PSL and PL. According to the PersL decay curve, all the samples exhibit no luminescence after ceasing UV-light irradiation for ~5 min (Supplementary Fig. S6a, b). However, the luminescence can be revived when the 808 nm NIR laser is turned on, even lasting for ~40 min (Supplementary Fig. S6c, d). These results indicate the existence of trap centres with a wide energy distribution in glass matrix/LiGa_5_O_8_ NCs for storing irradiation energy and the major traps are deep enough to be stable at room temperature. The optimized PSL performance is achieved in TGCs after annealing PG for 20–30 min (insets of Supplementary Fig. S6). Notably, the red emissive PSL signal for PG is rather weak (inset of Fig. 3a), whereas it increases substantially in TGCs due to the newly generated trap centres in glass, as revealed by the following trap analyses. In Fig. 3b, the PSL performance of LiGa_5_O_8_: Mn^2+^ NC-embedded TGC is examined in periodic excitation mode. Evidently, the sample can respond to NIR stimulation quickly, suggesting a fast information readout rate for ODS. The charging and detrapping of charge carriers are two dominant physical processes for PSL. In Fig. 3c, the optimum charging wavelengths for red and green PSL are determined as ~250 nm, viz. the peak of Mn^2+^–O^2−^ CTB (Supplementary Fig. S7 presents the creation of the PSL excitation spectrum). In Fig. 3d, only the 808 nm (photon energy: 1.54 eV) and 980 nm (photon energy: 1.27 eV) NIR lasers can release the trapped charge carriers, so the trap is deep enough.

**Fig. 4:**
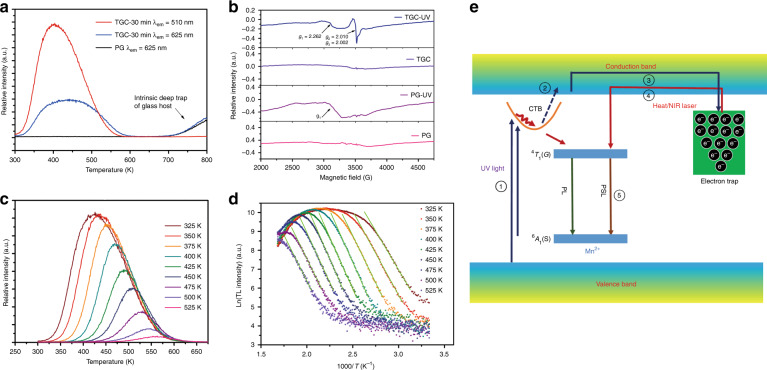
Trap analyses to reveal the PSL mechanism in the LiGa_5_O_8_: Mn^2+^ NC-embedded TGC. **a** TL glow curves of PG and TGC by monitoring green or red emissions. **b** EPR spectra of PG and TGC before/after 254 nm UV-light irradiation. **c** TL glow curves of TGC pre-excited by 254 nm UV light at various temperatures for 5 min (*λ*_em_ = 510 nm). In each TL measurement, the charged sample underwent quick cooling to 300 K and then heating at a rate of 1 K/s to 673 K. **d** Corresponding initial rise analyses on each TL glow curve of **c**. **e** Schematic illustration of the possible PSL mechanism for the LiGa_5_O_8_: Mn^2+^ NCs in glass, showing electron ① excitation, ② photoionization, ③ trapping, ④ release and ⑤ recombination

The trap property is key to the PSL performance^50^. To obtain an in-depth understanding of this phenomenon, the thermoluminescence (TL) technique is employed, aiming to monitor the detrapping behaviours of the immobilized charge carriers in defect centres as the temperature increases. The supplied thermal energy going beyond the thermal barrier, i.e., the energy of the trap depth (relative to the energy band of the host), produces PersL (whose colour is tunable in the present case; Fig. 2a) due to the released and recombined charge carriers responsive to heat. In Fig. 4a, the recorded TL spectrum for PG (*λ*_em_ = 625 nm) shows a TL peak higher than 800 K (reaching the limit of the thermal stage), suggesting the existence of intrinsic defect centres with ultradeep depth in the bandgap of the glass matrix. Upon glass crystallization, an additional broadband in the region of 300–600 K appears by monitoring the red emission (*λ*_em_ = 625 nm) and there is only a relatively narrowed band peaking at ~400 K by monitoring the green one (*λ*_em_ = 510 nm), both of which confirm the generation of fresh traps during transformation from amorphous to crystalline. We could make a preliminary postulation that the fresh traps responsible for the red and green PersL come from defects at the glass-crystalline interface (e.g., dangling bonds in the glass network) and intrinsic defects in LiGa_5_O_8_: Mn^2+^ NCs (e.g., oxygen vacancies, anti-site defects, and/or defect clusters), respectively. Electron paramagnetic resonance (EPR) results show no distinguishable signal for PG and TGC before UV-light irradiation, whereas intense signals with *g*_1_ = 2.262 for PG and *g*_1_ = 2.262, *g*_2_ = 2.010, and *g*_3_ = 2.002 for TGC are detected after exposing the samples to 254 nm light for 5 min, implying that three kinds of defect centres contribute to the storage of charge carriers (Fig. 4b). This coincides well with the TL results. Evidently, glass crystallization brings about new defects, resulting from the self-limited growth of LiGa_5_O_8_: Mn^2+^ NCs in glass. Then, we performed a series of controlled experiments by fixing all variables except for the charging temperature (Fig. 4c and Supplementary Fig. S8a), also called the ‘thermal-cleaning method’^51^, to determine the trap distribution in the LiGa_5_O_8_: Mn^2+^ NC-embedded TGC, wherein the charge carriers are gradually swept away from shallower traps as the temperature increases. With the aid of ‘initial rising analysis’^52^ (Supplementary Note 3, Fig. 4d and Supplementary Fig. S8b), the depths of the newly generated traps in TGC are evaluated to be 0.85–1.27 eV (LiGa_5_O_8_: Mn^2+^ NCs) and 0.89–1.64 eV (glass). Notably, to avoid the inaccuracy of the TL measurement brought by the thermal quenching effect, all the TL spectra were corrected by the curves of the temperature-dependent emission intensity at different wavelengths (Supplementary Fig. S9).

A possible PSL mechanism for the precipitated LiGa_5_O_8_: Mn^2+^ NCs in glass is proposed, as schematically illustrated in Fig. 4e. Upon irradiation by UV light, the electrons are first promoted to Mn-O CTB, delocalized to the CB of the host via photoionization and then are captured by traps with a continuous energy distribution^53^. A small part of electrons in shallow traps is soon depleted after ceasing the excitation source, producing PersL. The major electrons in deep traps have to be released under the stimulation of an NIR laser or heat^54–56^.

### Demonstration of application for 3D optical information storage

**Fig. 5:**
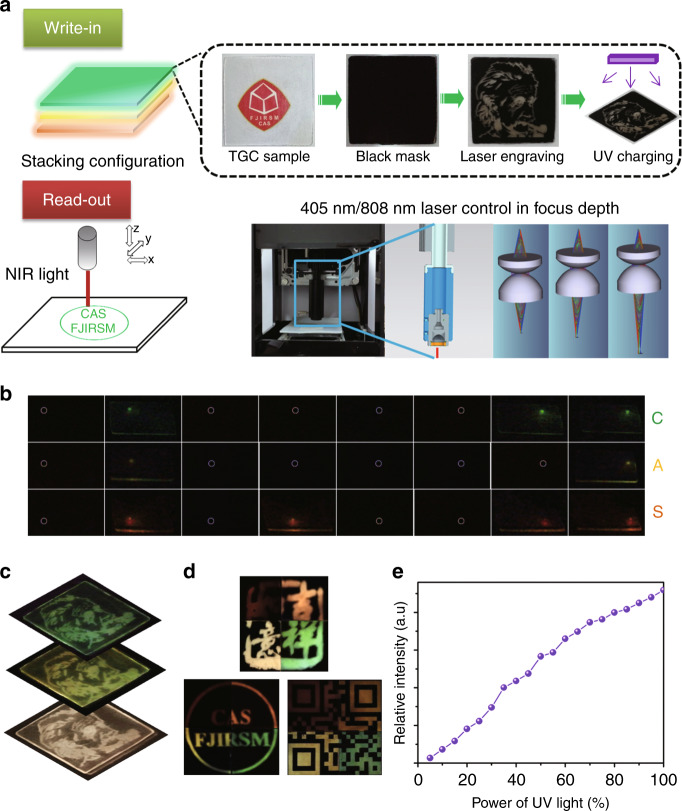
Demonstration experiment for 3D ODS with wavelength/intensity multiplexing. **a** Schematic illustration of the multilayer TGC-configured ODS medium and the write-in/readout process for optical information. **b** The 3D optical data readout using an 808 nm NIR laser (power density: 1.3 W/mm^2^) to show the encoded ‘C’, ‘A’ and ‘S’ of the binary system (the photos are taken through a 750 nm short-pass filter). **c** The 3D optical readout of the encoded images of Einstein in different layers with the aid of heat (150 °C). **d** Wavelength multiplexing by combining four TGC species with various heat treatment times into one system, where the multicolour-encoded Chinese characters with the meaning of ‘good fortunes’, English characters, and QR code can be read out by heating. **e** Demonstration of the intensity multiplexing by varying the input UV irradiation power. Note: the time lags set to take photographs are 3 s for heating and 1 s for turning on the 808 nm laser

The highly ordered nanostructure of LiGa_5_O_8_: Mn^2+^ NC-embedded PSL TGCs makes nanoscale optical resolution possible (there are some realistic challenges that may weaken the ability of this material to be applied in achieving nanoscale resolution) and enables a reduction in the error rate when applied to ODS. Except for that, the other great progress is the expanded dimension from 2D to three-dimensional (3D), considering that the volumetric ODS can improve the storage capacity to a great extent. As a proof-of-concept experiment, TGCs with green, yellow, and red PSLs are assembled in a stacking configuration (each TGC layer is polished to a thickness of 500 μm). Data write-in is achieved by initially masking the TGC plate by black ink, then employing a 405 nm blue laser driven by a computer programme to ablate the black ink at the set points and finally exposing the engraved patterns to a 254 nm UV lamp (Fig. 5a). Thereafter, the remaining black ink was removed with ethanol. By manually adjusting the focus depth of the laser beam, 3D write-in is realized (the custom-made focusing apparatus with simulation result is shown in the bottom right of Fig. 5a). Notably, the write-in procedure can be simplified by directly using a 254 nm laser engraving machine, which was not available in our lab. The data readout is achieved by an 808 nm NIR laser scanned progressively in bit-by-bit mode. One demonstration experiment is performed by encoding ‘C’, ‘A’ and ‘S’ of the binary system (CAS is the abbreviation of the Chinese Academy of Science), i.e., (01000011), (01000001) and (01010011), respectively, into different TGC layers (Supplementary Fig. S10a). The data decoding results show PSL in the encoded points (representing ‘1’) and no PSL in the blank points marked by white circles (representing ‘0’), as presented in Fig. 5b. The 3D ODS in the multilayer TGC can also be demonstrated by heating the respective layers to show TLs with different colours for the engraved pattern of Einstein (Supplementary Fig. S10b and Fig. 5c).

## Discussion

We report a new kind of ODS medium, TGC-containing PSL LiGa_5_O_8_: Mn^2+^ NCs, fabricated via thermally driven in situ glass crystallization. Microstructure analyses disclose a highly ordered nanostructure in an amorphous glass matrix that ensures the high transparency of the material for photonic applications. Spectroscopic studies reveal the variation in the coordination environment of Mn^2+^ before/after glass crystallization responsible for the adjustable PSL colour. With the aid of TL and EPR techniques, the relationship between trap properties and PSL performance is well established. A proof-of-concept experiment demonstrates the 3D volumetric ODS encoded/decoded by a UV-/NIR laser in bit-by-bit mode.

We evaluated the theoretical memory density of the developed PSL TGC 3D-ODS medium. Ideally, if we perform data encoding based on a confocal microscopy system (as that in ref. ^51^) with a 254 nm write-in laser diode and a common 0.85 NA focusing lens, the lateral and axial resolutions are Δ*r* = 0.4*λ*_0_/NA = 119 nm and Δ*z* = 1.4*λ*_0_/(NA)^2^ = 492 nm, respectively, and so the volume of a voxel is ~7.0 × 10^−15^ cm^3^ (translating to ~130 Tbit/cm^3^). One can expect a higher ODS density when using advanced far-field super-resolution optical microscopy to break the optical diffraction limit.

Wavelength- and intensity multiplexing have been proposed to further increase the storage capacity of ODS media^33,57^. In the studied PSL material, wavelength multiplexing can be realized by combining different TGC species with various heat treatment times into one system (Supplementary Fig. S10c–e and Fig. 5d), and intensity multiplexing is attained by changing the input UV irradiation power (Supplementary Fig. S11 and Fig. 5e). It is worth mentioning that the intensity could be perceived as a new dimension, where the greyscale encoding in each voxel enables the storage of information transform from a binary system to a multibinary system^58^.

In addition, LiGa_5_O_8_: Mn^2+^ NC-embedded PSL TGC as an ODS medium is notable for its high-security level. As seen from Supplementary Movie S1, the encrypted TGC with no visually discernible information trace decrypts only in two specific conditions: heating to >150 °C or irradiating with NIR light (using a laser beam expander). Moreover, in comparison with the previously reported 2D ODS on the surface of the medium, the optical data are preserved inside the bulk TGC more safely. In Supplementary Fig. S12, we show that the storage capacity, reflected as the integrated area under the TL spectrum, remains almost unchanged after experiencing 30 write-in/readout cycles, demonstrating good erasable–rewritable ability. This attribute benefits from the excellent thermal resistance of the robust glass matrix.

This work provides innovative thoughts and offers clear guidance on designing PSL materials for big data storage, hopefully bringing a renaissance to classical PSL materials.

## Materials and methods

### Preparation of LiGa_5_O_8_: Mn^2+^ TGCs

The PG with the nominal composition (in mol%) of SANGL was prepared via the conventional melt-quenching route. The analytical-grade reagents of SiO_2_ (Sinopharm Chemical Reagent Co., Ltd), Al_2_O_3_ (Sinopharm Chemical Reagent Co., Ltd), Na_2_CO_3_ (Sinopharm Chemical Reagent Co., Ltd), Ga_2_O_3_ (Nanjing Xinuo Tech. Co., Ltd), Li_2_CO_3_ (Sinopharm Chemical Reagent Co., Ltd) and MnCO_3_ (Sinopharm Chemical Reagent Co., Ltd) were weighed, mixed and ground as raw materials in an agate mortar. The obtained mixtures were then placed in a sealed alumina crucible and melted at 1640 °C for 30 min under ambient atmosphere. Subsequently, the glass melt was poured into a 300 °C preheated copper mould and annealed at 450 °C for 5 h to relinquish the internal stress. Ultimately, the obtained bulk glasses were cut into square coupons, polished and heat treated at 750 °C for different periods of time to induce crystallization of the LiGa_5_O_8_ nanophase.

### Characterization

XRD patterns of PG and TGC were characterized by a powder diffractometer (Rigaku, Miniflex600) at a scanning rate of 2.5°/min and a step size of 0.02° (Cu K_α_ radiation, *λ* = 0.154 nm). DSC (Netzsch, STA449F3) was carried out by heating ~20 mg of PG grains in an air atmosphere (α-Al_2_O_3_ crucible) at a heating rate of 10 °C/min. Microstructure characterization was performed by TEM (ThermoFisher Talos F200X), which operated at 200 kV. Steady-state, PersL/PSL and TL spectra were measured by a spectrophotometer (Edinburgh Instruments, FS920) equipped with a 450 W xenon lamp as the excitation source and a photomultiplier tube (R943-02, Hamamatsu) as the detector. The kinetic scanning mode of FS920 was utilized to record the PersL/PSL decay curve and the TL spectrum. To measure the PSL decay curve, an 808 nm NIR laser was used as the pumping source, which can be operated either in continuous or periodic mode. For the TL test, a cooling/heating stage (Linkam THMS600E) was used as the sample holder. X-band EPR spectra were obtained using an EPR spectrometer (Bruker, ELEXSYS E500) at a frequency of 9.826 GHz.

## Supplementary information

supporting information

Movie S1
