# Supplementary material for: High-security-level multi-dimensional optical storage medium: nanostructured glass embedded with LiGa5O8: Mn2+ with photostimulated luminescence
Source: Light Sci Appl. 2020 Feb 18;9:22. doi: 10.1038/s41377-020-0258-3 (PMC7028715; doi:10.1038/s41377-020-0258-3)
Supplement: Supplementary file 1 — supporting information [file 41377_2020_258_MOESM1_ESM.docx]

Supporting Information for

High-Security-Level Multi-dimensional Optical Storage Medium: Nanostructured Glass Embedded with LiGa_5_O_8_: Mn^2+^ with Photostimulated Luminescence

Shisheng Lin, ^a,b^ Hang Lin, ^a,*^ Chonggeng Ma,^c^ Yao Cheng, ^a^ Sizhe Ye, ^a,d^ Fulin Lin, ^a,d^ Renfu Li, ^a^ Ju Xu, ^a^ Yuansheng Wang ^a,*^

E-mail: [lingh@fjirsm.ac.cn](mailto:lingh@fjirsm.ac.cn); E-mail: [yswang@fjirsm.ac.cn](mailto:yswang@fjirsm.ac.cn);

^a^ Key Laboratory of Optoelectronic Materials Chemistry and Physics, Key Laboratory of Design and Assembly of Functional Nanostructures, Fujian Institute of Research on the Structure of Matter, Chinese Academy of Sciences, Fuzhou, Fujian, 350002 (P. R. China)

^b^ University of Chinese Academy of Sciences, Beijing, 100049 (P. R. China)

^c^ CQUPT-BRU Innovation Institute, Chongqing University of Posts and Telecommunications, Chongqing 400065, (P.R. China)

^d^ Xiamen Institute of Rare-earth Materials, Haixi Institutes, Chinese Academy of Sciences, Xiamen, Fujian, 361000 (P. R. China)

**Supplementary Note 1:**

According to the Mahadevan’s, Xia’s and Lin’s results,[^1-4^](#_ENREF_1) ∆T=T_c_-T_g_ is a useful indication to estimate the devitrification tendency during heat treatment for glass. ∆T~100 ℃ is a suitable value to achieve controllable process of nucleation and crystal growth. The larger ∆T indicates that the glass is unable to crystallize while annealing them at any temperatures between T_g_ and T_c_ even for a very long duration. If this parameter is too small, uncontrollable nucleation and crystal growth (self-crystallization process) would occur easily, which is against controllability. In this case, the ∆T value (79 ℃) is close to 100 ℃, indicating that it is favorable to realize controllable nucleation and crystal growth during heat treatment.

**Supplementary Note 2:**

Tanabe-Sugano (T-S) diagrams can be used to describe the spectral characteristics of Mn^2+^ ions in different environments. By solving the T-S matrix, the eqn(1) is acquired:

$B=\frac{94\alpha+\sqrt{49(T_{2}-T_{1})^{2}-768\alpha^{2}}}{49}$ (1)

where T_1_ and T_2_ are energies of ^6^A_1_(S)→^4^E(G) and ^6^A_1_(S)→^4^E(D) transitions derived from the excitation spectra, α represents the Tress correction coefficient and set to be 76 cm^-1^.[^5^](#_ENREF_5)^,^[^6^](#_ENREF_6) The B values are then calculated to be 767 cm^-1^ for Mn(VI) in glass and 780 cm^-1^ for Mn(IV) in LiGa_5_O_8_ NCs, respectively. Based on the Mn(VI) emission energy of 16000 cm^-1^ and the Mn(IV) one of 19608 cm^-1^, 10Dq are determined to be 11382 cm^-1^ and 3567 cm^-1^, respectively, according to the T-S diagram. It is noteworthy that the tetrahedral splitting energy of Mn^2+^ is 4/9 that of the octahedral splitting energy.

**Supplementary Note 3:**

An initial rising method was applied to the measured TL curves to reveal the shallowest occupied electron trap depth (E). This method assumes that the concentration of trapped electrons on the low-temperature side of a TL glow curve remains relatively constant, therefore the TL intensity (I(T)) should be independent on the TL kinetics and can be approximately expressed as[^7^](#_ENREF_7):

$I(\mathrm{T})=C\exp(\frac{-E}{kT})$ (1),

where C is the constant including the frequency factor *s*, and *k* the Boltzmann constant. By plotting the TL curves as ln(I) *v.s.* (1000/T), the shallowest trap depth E can be determined by the slope of a fitted straight section at the low-temperature side.


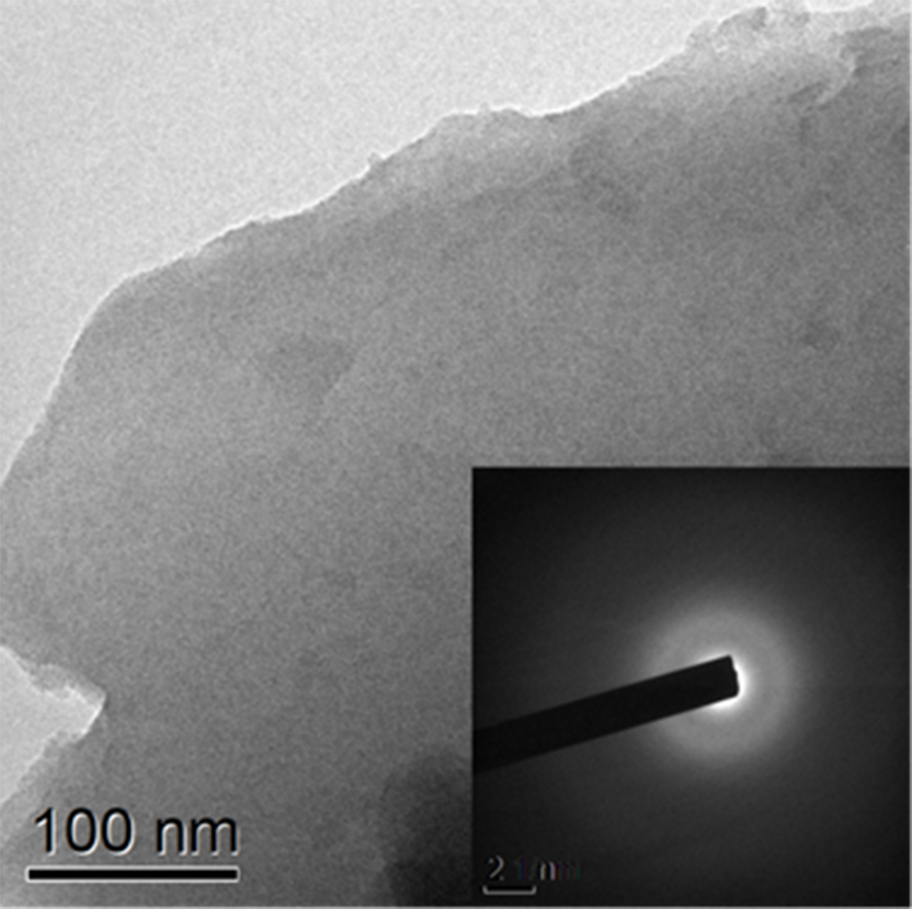


**Figure S1.** TEM image for the PG sample; the inset shows the corresponding SAED pattern with a halo ring.





**Figure S2.** Transmittance spectra of the PG and TGC heat-treated at different durations (sample thickness: 2.0 mm). The transmittance in the visible region remains higher than 70% after glass crystallization.


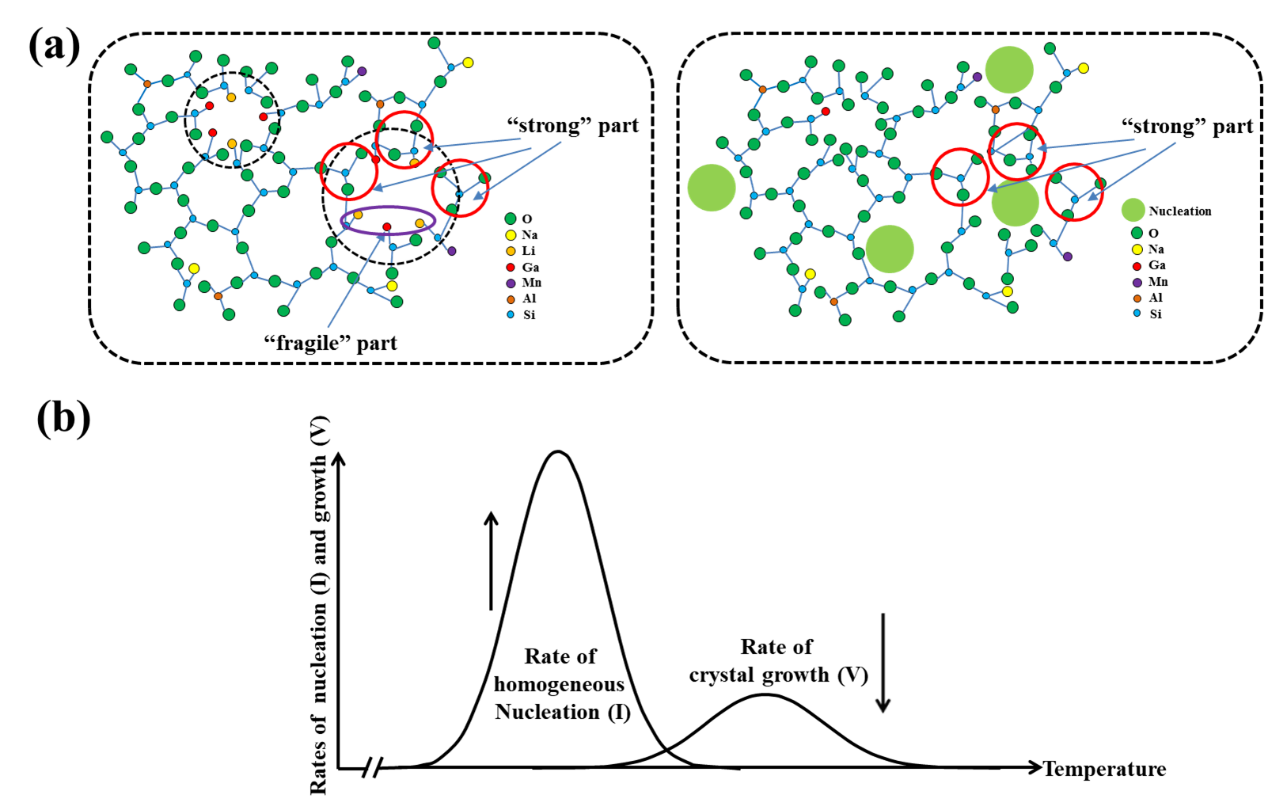


**Figure S3.** Schematic illustration of the self-limited growth of LiGa_5_O_8_: Mn^2+^ nanocrystals in glass.





**Figure S4.** The PL spectra of PG and TGC-10 min samples measured at 10 K.


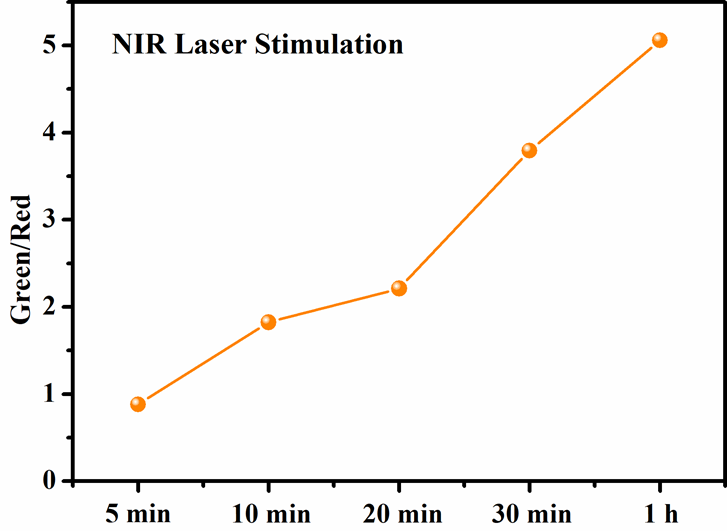

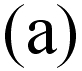

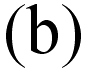

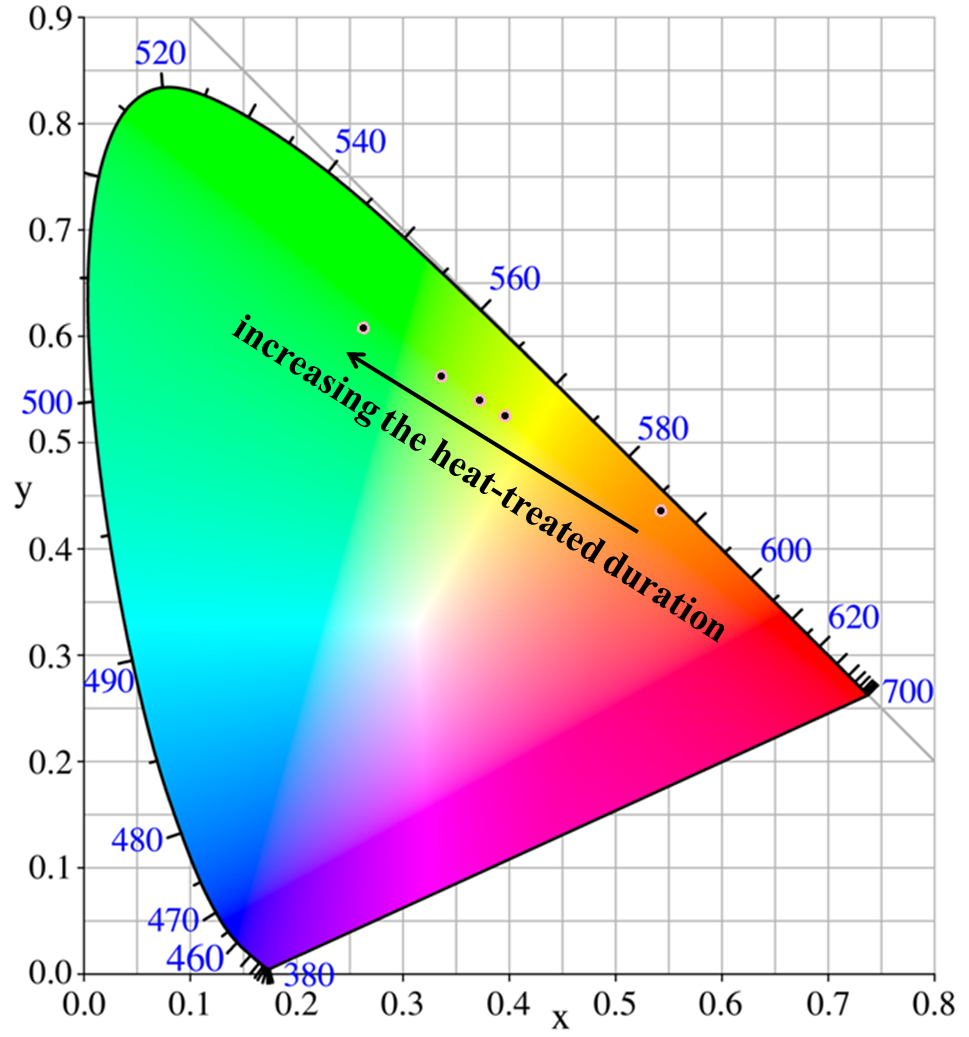


**Figure S5.** (a) The green/red ratio of PSL in the LiGa_5_O_8_: Mn^2+^ TGCs with various heat-treatement durations by NIR laser stimulation. (b) Corresponding color coordinates in the Commission Internationale de L'Eclairage 1931 diagram.


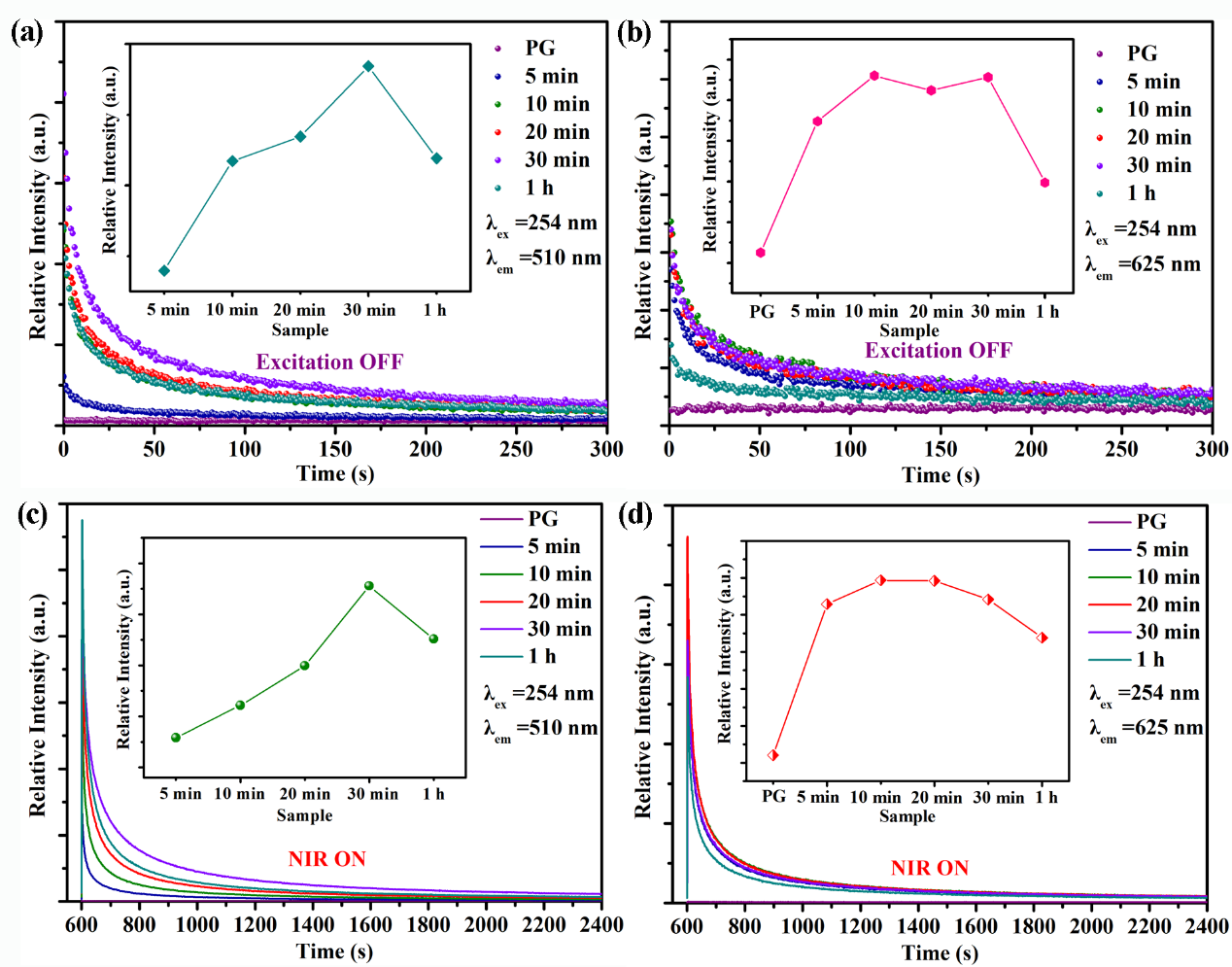


**Figure S6.** PersL (a-b) and PSL (c-d) decay curves of the PG and the TGC samples by monitoring at 510 nm and 625 nm emissions; the insets show the integrated emission intensities of samples under the corresponding decay curves. For both of the PersL and PSL tests, the samples were pre-irradiated by 254 nm UV light for 5 min. The 808 nm NIR laser in a continuous mode was then employed to examine the PSL performance.


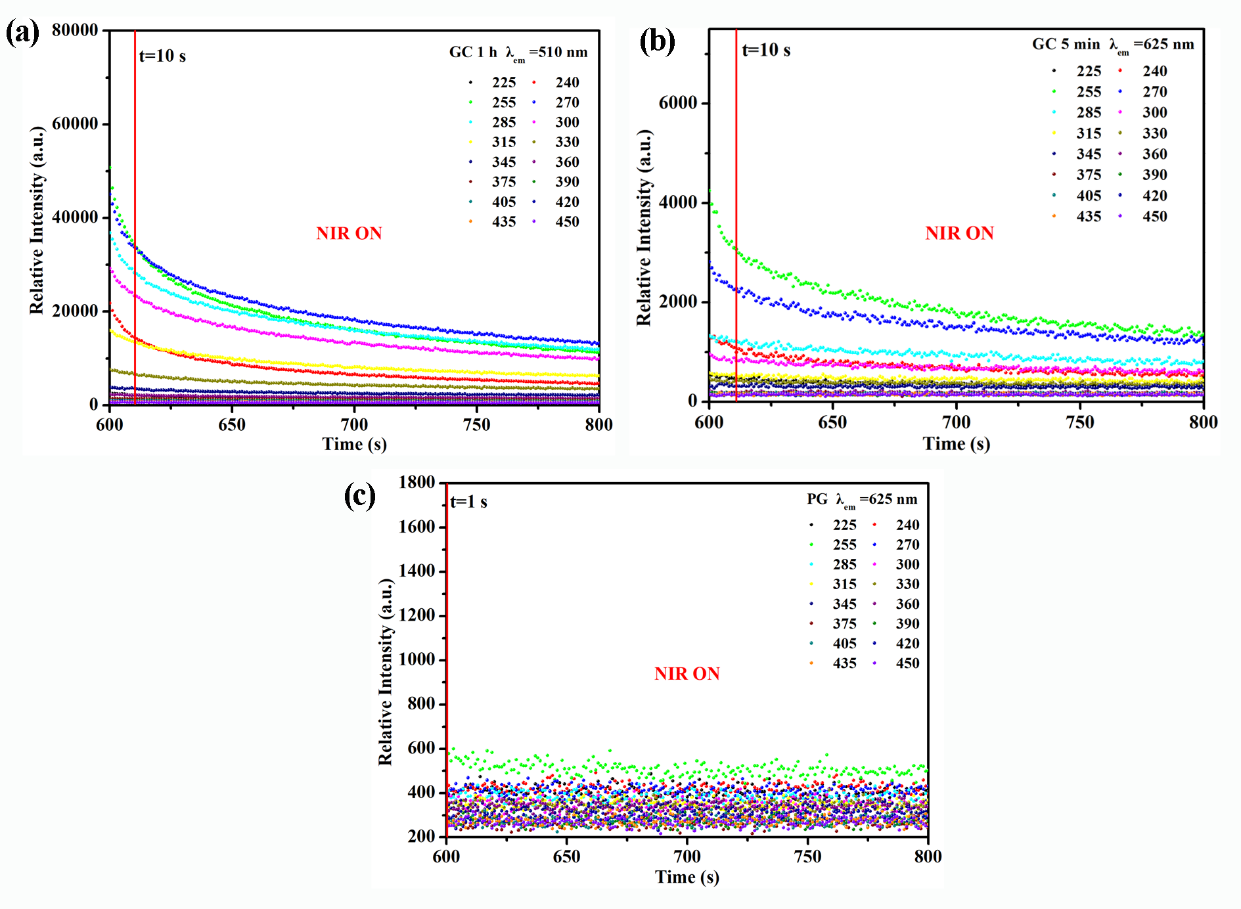


**Figure S7.** The creation of PSL excitation spectra by measuring the PSL decay curves for (a) TGC 1 h sample (λ_em_=515 nm), (b) TGC 5 min sample (λ_em_=625 nm), and PG sample (λ_em_=625 nm), under different charging wavelengths. All the samples are pre-irradiated by 254 nm light for 5 min, delayed for 5 min, and then continuously irradiated by 808 nm laser. The luminescence intensities acquired at the time of 10 s (for PG sample, this duration is set 1 s) after turning on 808 nm laser were recorded for creation of the PSL excitation spectrum.


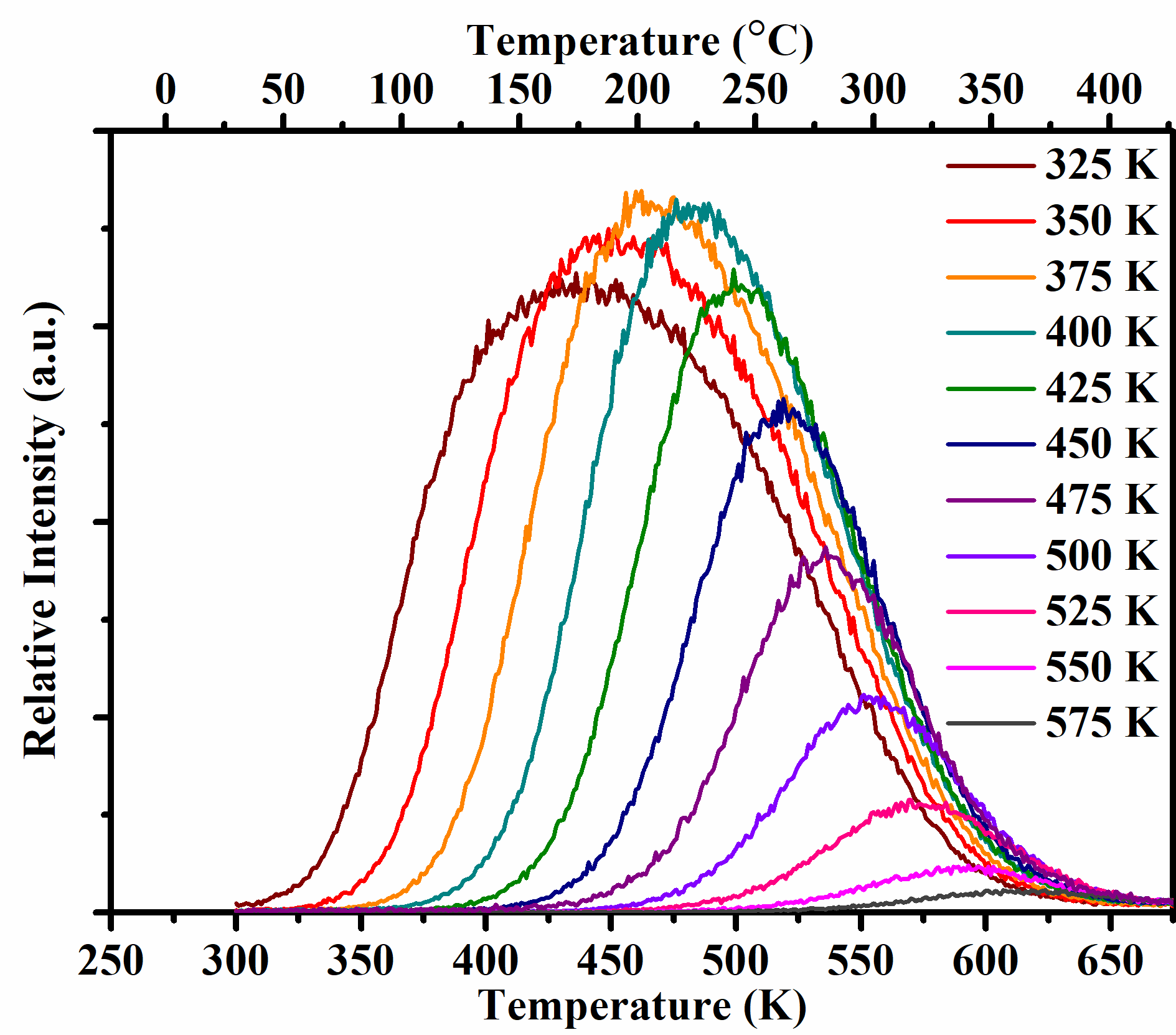

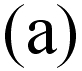





**Figure S8.** (a) TL glow curves of TGC-10 min sample pre-excited at various temperatures (λ_ex_=254 nm, heating rate: 1 K s^-1^, λ_em_=625 nm). (b) Corresponding initial rise analyses on the TL glow curves in (a).


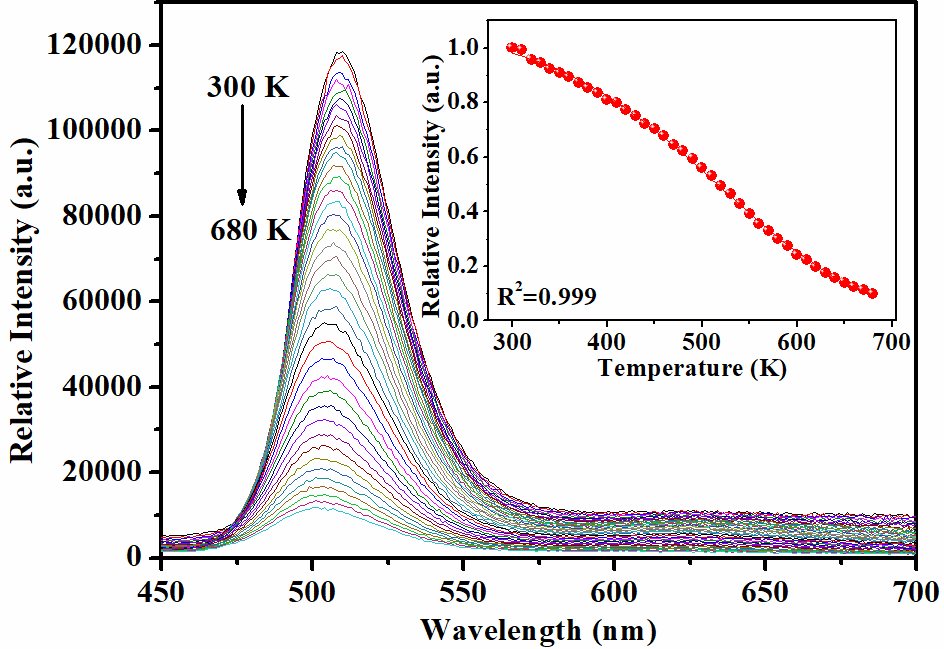

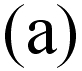

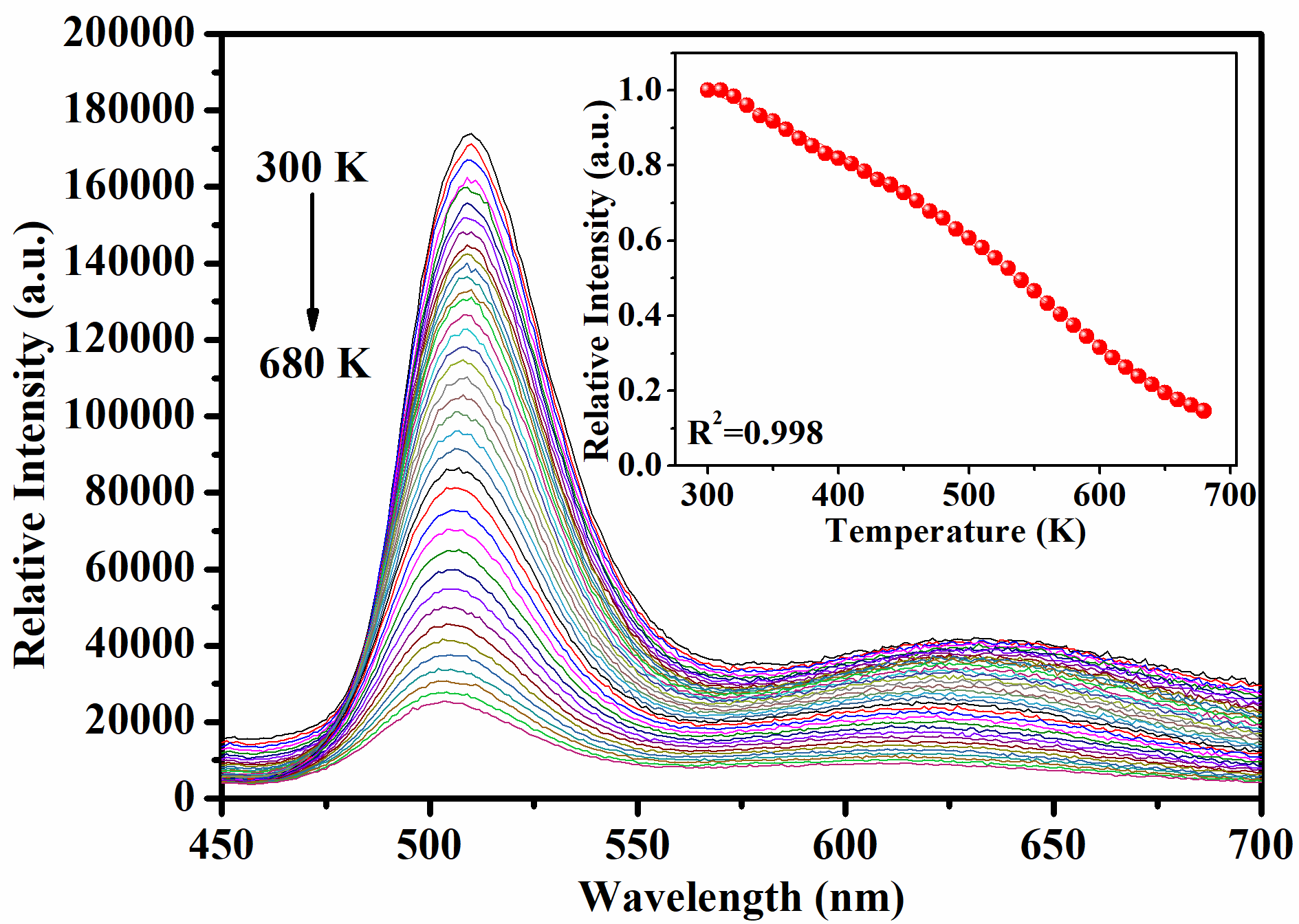

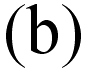


**Figure S9.** Temperature-dependent PL spectra in (a) TGC-1 h and (b)TGC-10 min samples under 254 nm excitation. The inset shows the relative intensity as a function of temperature.

**
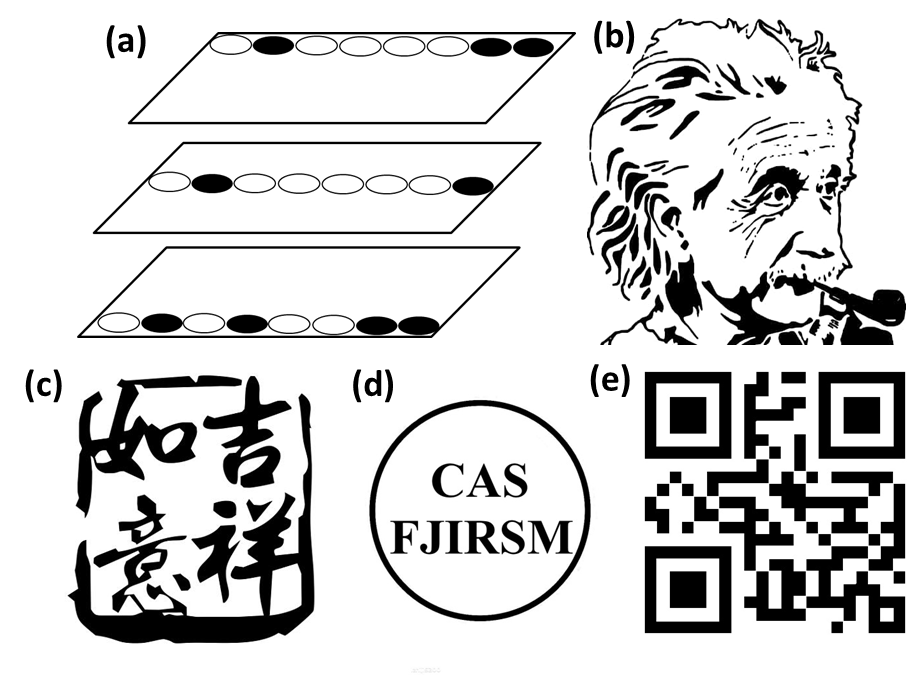
**

**Figure S10.** Original patterns showing (a) “C”, “A”, and “S” of the binary data system in different TGC layers, (b) picture of Einstein, (c) Chinese characters with meaning of “good fortunes”, (d) English characters, and (e) QR code for optical information recording. The recording laser outputs only on the black points.





**Figure S11.** PSL decay curves of the TGC charged by 254 nm UV light with different power (λ_em_=510 nm, power of 808 nm laser: 1.0 W)


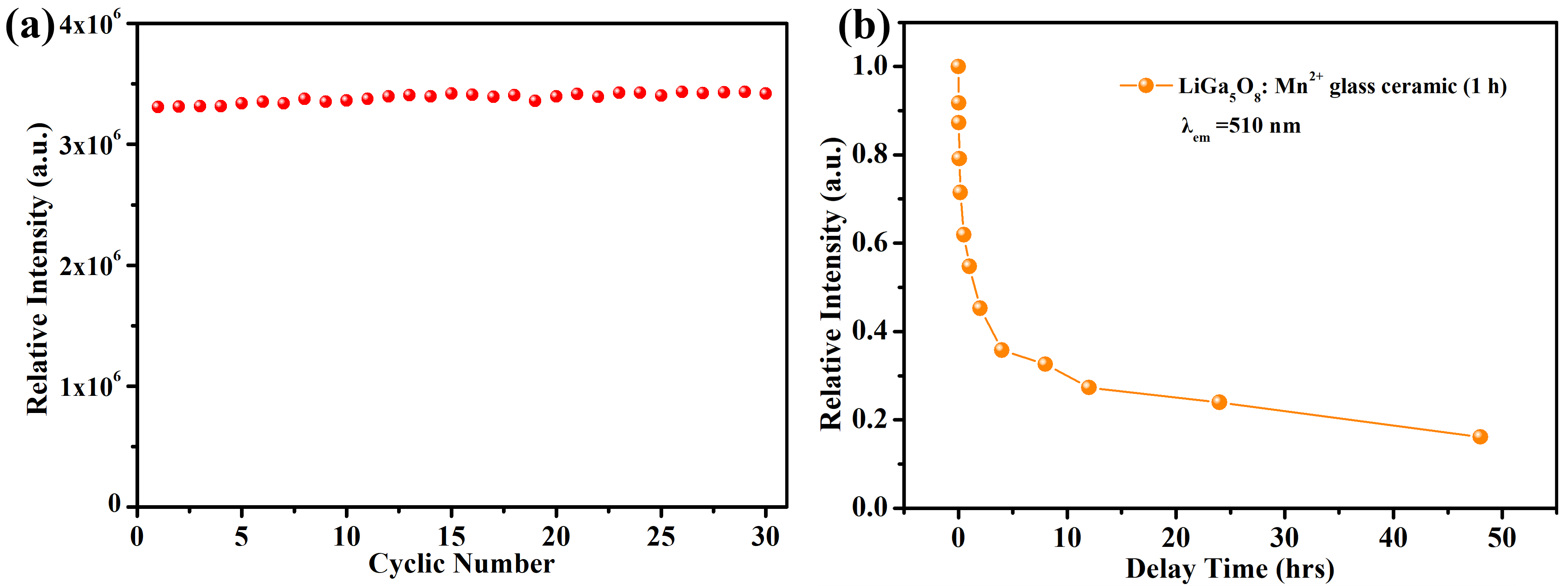


**Figure S12.** Erasable-rewritable ability test on the TGC sample for 30 write-in/read-out cycles. In each cycle, the TGC sample was irradiated by 254 nm light for 5 min (wirte-in), delayed for 300 s at 300 K, heated to 673 K at a rate of 1 K s^-1^ (read-out), and finally cooled back to 300 K. During heating, TL glow curves are recorded.

**Reference**

1 Mahadevan, S., Giridhar, A. & Singh, A. Calorimetric measurements on as-sb-se glasses. *J. Non-Cryst. Solids* **88**, 11-34 (1986).

2 Xia, F. *et al.* Glass formation and crystallization behavior of a novel GeS_2_-Sb_2_S_3_-PbS chalcogenide glass system. *J. Am. Ceram. Soc.* **89**, 2154-2157 (2006).

3 Lin, C. *et al.* Second-order optical nonlinearity and ionic conductivity of nanocrystalline GeS_2_-Ga_2_S_3_–LiI glass-ceramics with improved thermo-mechanical properties. *Phys. Chem. Chem. Phys.* **12**, 3780-3787 (2010).

4 Lu, P., Zhang, Y. & Li, J. Thermal and structural analysis of 40La_2_O_3_-10Nb_2_O_5_-(50-x)Al_2_O_3_-xBaO glasses. *J. Non-Cryst. Solids* **522**, 119558 (2019).

5 Rao, J. L. & Purandar, K. Electronic absorption spectrum of Mn^2+^ ions doped in diglycine barium chloride monohydrate. *Solid State Commun.* **37**, 983-986 (1981).

6 Mehra, A. K. Trees correction matrices for d5 configuration in cubic symmetry. *J. Chem. Phys.* **48**, 4384-4386 (1968).

7 Bos, A. Theory of thermoluminescence. *Radiat. Meas.* **41**, S45-S56 (2006).
